# Starch-Derived Superabsorbent Polymer in Remediation of Solid Waste Sludge Based on Water–Polymer Interaction

**DOI:** 10.3390/polym15061471

**Published:** 2023-03-16

**Authors:** Juan Matmin, Salizatul Ilyana Ibrahim, Mohd Hayrie Mohd Hatta, Raidah Ricky Marzuki, Khairulazhar Jumbri, Nik Ahmad Nizam Nik Malek

**Affiliations:** 1 Department of Chemistry, Faculty of Science, Universiti Teknologi Malaysia UTM, Johor Bahru 81310, Johor, Malaysia; 2 Centre for Sustainable Nanomaterials, Ibnu Sina Institute for Scientific and Industrial Research, Universiti Teknologi Malaysia UTM, Johor Bahru 81310, Johor, Malaysia; 3 Centre of Foundation Studies, Universiti Teknologi MARA Cawangan Selangor, Kampus Dengkil, Dengkil 43800, Selangor, Malaysia; 4 Centre for Research and Development, Asia Metropolitan University, Johor Bahru 81750, Johor, Malaysia; 5 Department of Fundamental and Applied Sciences, Universiti Teknologi PETRONAS, Seri Iskandar 32610, Perak, Malaysia; 6 Department of Biosciences, Faculty of Science, Universiti Teknologi Malaysia UTM, Johor Bahru 81310, Johor, Malaysia

**Keywords:** superabsorbent polymer, water–polymer interaction, solid waste sludge treatment, starch biopolymer, water absorbency

## Abstract

The purpose of this study is to assess water–polymer interaction in synthesized starch-derived superabsorbent polymer (S-SAP) for the treatment of solid waste sludge. While S-SAP for solid waste sludge treatment is still rare, it offers a lower cost for the safe disposal of sludge into the environment and recycling of treated solid as crop fertilizer. For that to be possible, the water–polymer interaction on S-SAP must first be fully comprehended. In this study, the S-SAP was prepared through graft polymerization of poly (methacrylic acid-co-sodium methacrylate) on the starch backbone. By analyzing the amylose unit, it was possible to avoid the complexity of polymer networks when considering S-SAP using molecular dynamics (MD) simulations and density functional theory (DFT). Through the simulations, formation of hydrogen bonding between starch and water on the H06 of amylose was assessed for its flexibility and less steric hindrance. Meanwhile, water penetration into S-SAP was recorded by the specific radial distribution function (RDF) of atom–molecule interaction in the amylose. The experimental evaluation of S-SAP correlated with high water capacity by measuring up to 500% of distilled water within 80 min and more than 195% of the water from solid waste sludge for 7 days. In addition, the S-SAP swelling showed a notable performance of a 77 g/g swelling ratio within 160 min, while a water retention test showed that S-SAP was capable of retaining more than 50% of the absorbed water within 5 h of heating at 60 °C. The water retention of S-SAP adheres to pseudo-second-order kinetics for chemisorption reactions. Therefore, the prepared S-SAP might have potential applications as a natural superabsorbent, especially for the development of sludge water removal technology.

## 1. Introduction

Over the past decades, industrial solid waste sludge has primarily been disposed of in open landfills, causing hazardous contamination of water resources with toxic pollutants [1,2]. When solid waste sludge is exposed to rain, the environmental problem becomes severe. This is because toxic pollutants start to leach out of the sludge, spreading into soil and underground water [3,4,5]. To reduce the increasing number of open landfills for waste treatment, the use of incinerator systems is a more preferable method of getting rid of industrial wastes [6]. In the process, ash, flue gas, and heat are produced, causing air pollution as a secondary environmental issue [6,7,8,9]. Another method for treating solid waste sludge is membrane technologies, which require a long operating time and very high maintenance costs [10,11,12,13]. With prolonged use, the performance of the membrane system will decrease because of certain drawbacks such as compaction, fouling and scaling [14]. In light of sustainability and cost-effectiveness issues, the development of a more efficient solid waste sludge treatment system is urgently needed.

For more cost-effective waste management in industry, the removal of water and toxic metals from sludge to a dried non-hazardous solid is highly desirable to reduce environmental impact. In addition, treated sludge can be safely disposed of into the environment and reused as fertilizers [15]. In view of this, superabsorbent polymers (SAPs) have attracted attention for the treatment of industrial waste due to their high water absorption capacity [16,17]. For example, a petroleum-based SAP composed of acrylic acid and acrylamide is used to remove heavy metals from wastewater [18,19]. To form natural-based SAPs, grafting copolymerization strategies onto carboxymethylcellulose (CMC) [20,21,22] and the deacetylation process of chitin from crab, crayfish, and shrimp shells [23,24,25] have extensively been studied. Recently, Menceloğlu et al. (2022) developed SAP–halloysite nanotube (HNT) via free radical polymerization to enhance water retention capacities and rheological characteristics [26]. Nevertheless, the HNT filler in the SAP limits their application to solid waste sludge treatment and reduces their biodegradability. In comparison to petroleum-based SAPs, biopolymer SAPs, derived from natural resources such as cellulose, chitosan and carbohydrates, are more environmentally friendly and biodegradable [27,28].

In 2021, Chang and co-workers studied SAPs carbohydrates originating from various starches to demonstrate decent water capacity in agriculture usage [29]. Similarly, Sanders et al. (2021) used a combination of MD simulations and the grand canonical Monte Carlo (GCMC) method to study the effect of water on starch [30]. In this technique, the emulsifiers’ processes are limited to single-molecule host–guest binding in dilute solutions. Meanwhile, Zhiguang et al. (2022) demonstrated an unwound double-amylose helix, and enhanced the bending degree of starch molecules using MD simulation [31]. However, the MD simulation could only be performed at high temperatures that affect the conformation of the starch molecules. Evidently, the interaction between water and polymer on biopolymer SAP, particularly starch, can be difficult to correlate, either experimentally or through simulation.

Nonetheless, it is necessary to design an experiment to improve the water retention capacities of SAPs. The main problems with finding suitable biopolymers to prepare SAPs lie in the complexity of polymer networks. To tackle these problems, this work is the first investigation to be conducted on the starch-derived SAP (S-SAP) using MD simulations and density functional theory (DFT), based on their simplest polymer backbone, the amylose unit, to determine the water–polymer interactions. To support the simulation findings, the water retentions of S-SAP in distilled water and solid waste sludge were evaluated and confirmed using Fourier transform infrared (FTIR) spectroscopy. In order to fully explain the effect of water variations on hydrogen bond formation, starch’s involvement in producing a high water retention capacity is crucial. 

To the best of our knowledge, the study of this type of S-SAP for the treatment of solid waste sludge has not been reported elsewhere. It may be possible to use MD simulation to predict the specific interactions between water and other biopolymer-SAPs based on the original works presented here. By adopting these approaches, a brand new biopolymer backbone can be functionalized or grafted into excellent SAP materials without any risks of unwanted molecular interference and complicated experimental design.

## 2. Materials and Methods

### 2.1. Materials

The starch (C_6_H_10_O_5_)_n_, (p.a. 99%; CAS number: 9005-25-8 from Sigma Aldrich, St. Louis, MO, USA), methacrylic acid C_4_H_6_O_2_, (p.a. 99%; CAS number: 79-41-4 from Sigma Aldrich, St. Louis, MO, USA), ammonium persulfate (NH_4_)_2_S_2_O_8_, (p.a. 99%; CAS number: 7727-54-0 from Sigma Aldrich, St. Louis, MO, USA), N,N′-methylenebisacrylamide C_7_H_10_N_2_O_2_, (p.a. 99%; CAS number: 110-26-9 from Merck KGaA, Darmstadt, Germany), sodium dodecyl sulfate (CH_3_(CH_2_)_11_SO_4_Na, (p.a. 99%; CAS number: 151-21-3 from Sigma Aldrich, St. Louis, MO, USA), and p-octyl poly(ethylene glycol)phenyl ether (p.a. 99%; CAS number: 48145-04-6 from Sigma Aldrich, St. Louis, MO, USA) were purchased and used for the preparation of superabsorbent. The starch was used without further purification, while methacrylic acid was distilled under reduced pressure prior to usage. Meanwhile, the ammonium persulfate (APS) was recrystallized from water before usage. All other chemicals are of analytical grade and the solutions were prepared with distilled water.

In this study, the solid waste sludge is classified as the primary solid waste (SW 204) obtained as an offering from an undisclosed disposal facility of a polymer production plant located at Pasir Gudang, Johor, Malaysia. Prior to the collection of solid waste sludge, the sample was thickened in the waste treatment ponds by adding cationic polyacrylamide as the organic flocculants to remove water. Later, the separated solid waste sludge was allowed to flow over the filtration conveyor on a gravity belt, while the drained wastewater was discharged through the permeable slit of the belt. Consequently, the solid waste sludge entrapped high amounts of water primarily due to the presence of organic and inorganic mixture. The basic composition and physical characteristics of the solid waste sludge for the test are shown in Table 1. The sludge obtained from solid waste is used directly without any pretreatment.

### 2.2. Preparation of Starch-Derived Superabsorbent Polymer (S-SAP)

1.44 g of starch was added to 30 mL of distilled water in a flask equipped with a stirrer, condenser, and thermometer. The slurry was stirred continuously and gelatinized at 90 °C for 30 min, before being cooled to 40 °C. Afterward, 1.5 mg of sodium dodecyl sulfate (SDS) was dissolved in 2.0 mL of water, while 9.4 mg of organophosphate was added into the gelatinized starch solution. Upon vigorously stirring for 15 min, the volume of the slurry increased greatly, and foaming starch was obtained. In a different flask, 7.2 g of methacrylic acid was partially neutralized with 12.78 mL of 5 M NaOH solution, followed by the additions of 19.2 mg of N,N′-methylenebisacrylamide (MBA) and 124.8 mg of APS to the mixture. Subsequently, the solubilised mixture was poured into the foaming starch slurry. To complete the polymerisation reaction, the water bath was heated at 55 °C for 3 h. The formed gels were dewatered by immersing them into 100 mL of ethanol for 48 h. Finally, the excess ethanol was removed from the surface using a filter paper, and the samples were spread on a Petri dish for an overnight drying process at room temperature.

### 2.3. Characterization

Morphological properties were characterized using a field emission scanning electron microscope (FESEM), Hitachi SU8020 and 20 keV. Prior to FESEM imaging, the samples were coated with platinum (Pt) using a vacuum sputter coater. The elemental compositions in the prepared samples were analyzed by using energy dispersive X-ray (EDX) (Hitachi) analysis. The structural properties of the samples were characterized by X-ray diffraction spectroscopy (XRD) using a Rigaku with Cu anode (PAN analytical Co. X’Pert PRO) at 40 kV and 30 mA. The Fourier transform infrared (FTIR) spectroscopy was performed to investigate the functional groups and chemical bonding using an attenuated total reflectance Perkin Elmer spectrophotometer, and the wavelengths ranged from 4000 cm^−^^1^ to 650 cm^−^^1^. Both the solid and liquid samples had preset scan numbers of 8 and 16, respectively. A Mettler-Toledo model DSC STAR system was used to perform differential scanning calorimetry (DSC) thermogram. A certain amount of the sample was heated up to 400 °C at a heating rate of 10 °C/min. A Philips PW 1400 WD-XRF spectrometer, equipped with a W anticathode X-ray tube (50 kV, 25 mA), scintillation gas proportional counters in tandem and a LiF [200] crystal, was used for the X-ray fluorescence (XRF) analysis.

### 2.4. Density Functional Theory

For visualization of the compounds involved, GaussView 5 software was employed in this study. Meanwhile, calculation of the studied compound was performed by applying the density functional theory method on the B3LYP functional and 6-31G (d,p) basis set using Gaussian 16 software obtained from the Centre for Information Communication and Technology (CICT), Universiti Teknologi Malaysia.

### 2.5. Molecular Dynamics 

**Figure 1:**
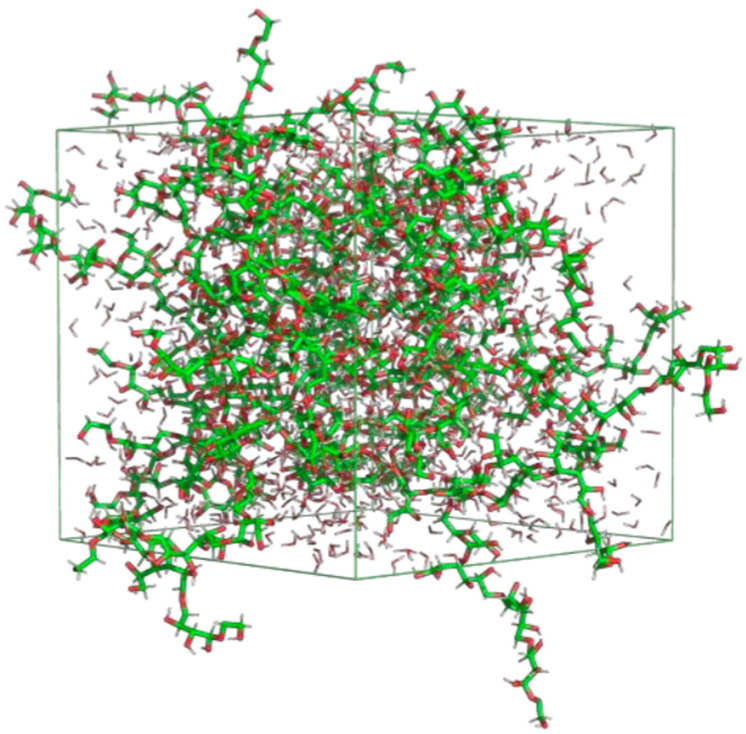
The cubic simulation box filled with amylose and water molecules.

To simulate the water–polymer condition, the model structure of the simplest form of starch based on linear polymer amylose was obtained from the Research Collaboratory for Structural Bioinformatics Protein Data Bank RCSB-PDB, through http://www.rcsb.org/ (accessed on 16 June 2020), with a PDB ID 5JIW. A cubic simulation box was used and the volume of the box was calculated based on a cut-off distance of 12 Å. In this molecular study, fifty molecules of amylose were randomly placed in a 64 nm^3^ simulation box with periodic boundary conditions (PBC) applied in all directions. The box was then filled with the requisite number of SPC models of water molecules. A GROMOS 56A6CARBO Force Field was deployed to represent the intra–inter-molecular potential of polymer and water. Figure 1 depicts the cubic simulation box filled with amylose and water molecules.

In computing the MD simulation, the following parameters were used. Primarily, the integration step of 2.0 fs was used. The non-bonded interactions were calculated up to 12 Å, and the long-range electrostatic interactions were adopted using particle mesh Ewald (PME) with a grid spacing of 1.2 Å and fourth-order interpolation. Neighbour searching was carried out up to 12 Å and updated every five steps. The bond lengths were constrained using LINCS. Temperature and pressure controls were implemented using the Berendsen thermostat and Berendsen barostat, respectively. The reference pressure was set to 1.0 bar, and a relaxation time of 2.0 ps was applied. The isothermal compressibility for pressure control was set to 4.5 × 10^−5^ bar^−1^. In addition, the heat was separated in two heat baths with a temperature coupling constant of 0.1 ps, while two-step energy minimization was performed with each of the respective energy systems minimized at 10,000 steps of the steepest descent, followed by 10,000 steps of conjugate gradients. The system was then introduced in the canonical ensemble (NVT) for 2000 ps. Two different temperatures, which were 298 and 323 K, were deployed for 10 ns during the production simulation.

The average root mean square deviation (RMSD) was calculated by fitting the simulated amylose against the initial X-ray crystal structure, while the radial distribution function (RDF) was determined between the residues’ center-of-mass (RES-COM) of water molecules around the amylose. The presence of the formation of hydrogen bonding between the two molecules can be confirmed, provided that the distance between the hydrogen atom and the acceptor is less than 0.35 nm and the angle formed by acceptor–donor-hydrogen is less than 30°. The hydrogen bonding interactions were calculated as an average. All the pictures shown were created using PyMol.

### 2.6. Absorption Properties

The absorption studies are conducted for the absorbency test on dry S-SAP in distilled water (as control) and the absorption of wastewater from sludge. In the respective absorption test, three replicates of ca. 1 g of dry S-SAP were prepared, placed in a nylon tea bag, and immersed in a 250 mL beaker with a sufficient amount of distilled water at room temperature to reach the saturation point of S-SAP. The difference in absorbency (Q_water_) of the SAPs was calculated using Equation (1).

(1) $$Q_{\mathrm{water}}\;=\frac{m_{f}-m_{i}}{m_{i}}\times 100\%$$

where *m_i_* is the initial weight of the S-SAP and *m_f_* is the final weight of the S-SAP.

For the wastewater absorption from sludge, three replicates of approximately 2 g of the dry S-SAP composites were prepared and placed in a nylon tea bag. In this part, a large amount of dry S-SAP was used in order to provide more surface area for the absorption of wastewater from solid sludge. Subsequently, the prepared tea bags were immersed in a beaker with a sufficient amount of SW 204 sludge at room temperature to reach swelling equilibrium. To calculate the absorbed wastewater from sludge, Equation (1) was used. 

### 2.7. Swelling Properties

Some 1 g of S-SAP was transferred into a nylon tea bag before immersing it in a beaker with a sufficient amount of distilled water at room temperature to reach the swelling equilibrium. The amount of water entrapped by S-SAP was weighed upon removing the excess water. The weight of the swollen S-SAP was recorded based on the swelling ratio, which corresponded with the size of the swollen S-SAP. The swelling ratio (*q*) was determined by using Equation (2):

(2) $$q_{\;}=\frac{M_{t}-M_{o}}{M_{o}}$$

where *M_t_* is the weight of the swollen SAP and *M_o_* is the weight of the dry S-SAP. The water retention of the swollen S-SAP was determined by heating them at different temperatures (60 °C, 70 °C, 80 °C, 90 °C and 100 °C) for 5 h. 

## 3. Results and Discussion

### 3.1. Synthesis of Starch-Derived Superabsorbent Polymer (S-SAP)

**Figure 2:**
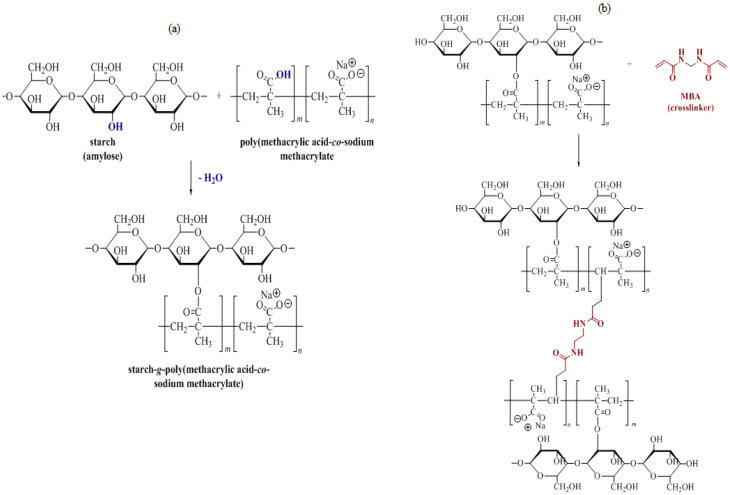
Synthetic procedures for S-SAP: (**a**) reaction of starch with poly(methacrylic acid-co-sodium methacrylate), (**b**) proposed cross-linking of grafted starch polymer.

In this study, the S-SAP was formed by graft polymerisation of poly(methacrylic acid -co-sodium methacrylate) as a monomer on the starch, in the presence of methylenebis-acrylamide (MBA) as a crosslinker and ammonium persulfate (APS) as a radical initiator. The neutralization of methacrylic acid took place when the monomer was grafted onto the starch network. At high temperatures, the initiator (APS) was notably present in the solution to decompose and produce sulphate anion radicals (SAR). This SAR would initiate the polymerization process by activating the monomer, and the active sites in the monomer would then act as free radical donors to the adjacent monomers prior to the propagation of the homo polymer. During the polymerization process, a polymer chain was created, with the end vinyl group in the cross-linker producing inter-penetrating, three-dimensional S-SAP containing an abundant number of free carboxyl groups. These reactions are shown schematically in Figure 2.

### 3.2. Determination on Water–Polymer Interaction of Starch Backbone 

**Figure 3:**
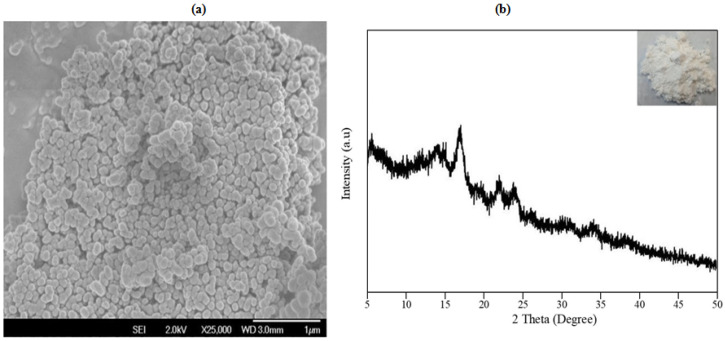
(**a**) The FESEM micrograph of potato starch; and (**b**) The XRD diffractogram of potato starch. Inset represents a photograph image of starch powder.

The study on the molecular structure of starch was conducted to understand the nature, mode of mechanism, and possible interaction of starch prior to the preparation of S-SAP. This study also determined the ability of starch to act as the backbone of a superabsorbent polymer. The complex structure of starch granules is the result of a combination of two main macromolecules of amylose and amylopectin, with the repeating units of both molecules known as D-glucan and glucopyranose. Notably, starch exists in the form of granules of different sizes and shapes. Typically, this type of starch demonstrates round and oval granules of various sizes, as shown in Figure 3a. In particular, starch possesses a semi-crystalline morphology with different degrees of crystallinity depending on their classes. Based on the XRD pattern shown in Figure 3b, the starch shows several intense peaks at 2θ of 5.5°, 15° and 17°. A few less intense peaks were also observed at 2θ of 23° and 24°. The observed diffraction patterns suggest that the respective starch has semi-crystalline properties. In addition, the broad diffraction peaks observed at the range of ca. 2θ of 10°–17° indicate the presence of small crystallite sizes. 

**Figure 4:**
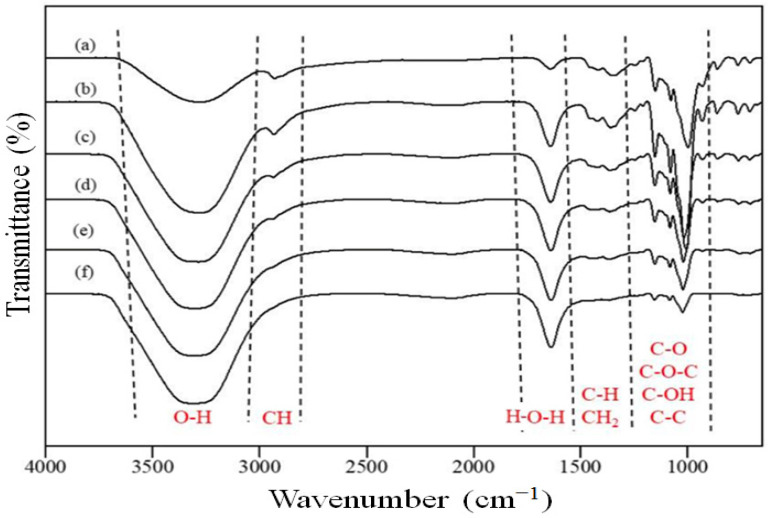
The FTIR spectra of starch in with the presence of different concentrations of water; (a) 0 mol, (b) 1 mol, (c) 2 mol, (d) 3 mol, (e) 4 mol and (f) 5 mol.

To determine the functional groups and effects of water–polymer interactions on the starch granules, FTIR spectroscopy measurements were carried out, as shown in Figure 4. The appearance of a wide peak at the region of 1200–800 cm^−1^ corresponded to the vibration band of glucan ring, which overlapped with the stretching mode of C-OH and the bending vibration of the C-O-C glycosidic band [32]. On the other hand, the peaks that appeared at the wavelengths of 1149 cm^−1^, 1077 cm^−1^ and 997 cm^−1^, [32,33] were attributed to the formation of C-O and C-C stretching, as well as the formation of C-OH [33,34]. Meanwhile, the peak observed at ca. 1638 cm^−1^ corresponded to the bending mode of H-O-H, which resulted from the consecutive addition of water. The presence of the respective peak is in agreement with the findings reported in the literature [34,35].

In this process, the formation of hydrogel is initiated as starch starts to gelatinize into a stable three-dimensional network. This is a crucial step to disrupt the crystalline structure of starch and to enable the strong interaction of the water–polymer chain. Moreover, the ability of starch to form hydrogen bonds with water is a crucial indication that the starch is capable of absorbing water efficiently. Basically, when starch is treated with water in excess, the crystalline structure of the starch’s double helices is affected due to the breakage of internal hydrogen bonding. Therefore, the water molecules will form a bonding with the exposed hydroxyl groups of amylose and amylopectin. During this process, the granular structure of starch begins to swell and its solubility in water increases. Consequently, the stretching vibration appears at ca. 1200–800 cm^−1^, which refers to the crystalline domains that contain the double helices of amylopectin side chains [36] becoming less significant as more water is added consecutively. However, it was observed that the FTIR spectra of respective crystalline domains became more intense and significant, as demonstrated by the stretching vibration at ca. 1200–800 cm^−1^. The significant increase might be due to the retrogradation process in starch, in which the broken double helical crystalline structure in amorphous starch molecules reformed after losing their water binding ability [37].

To further confirm the presence of the hydrogen bonding interaction between starch and water, the density functional theory (DFT) was also performed to model the dynamic structure of starch and water. From Figure S1a, the bond length of O11 and H11 after optimization using a basic DFT/6-31G (d,p) level provides the value of 1.85 Å, indicating the hydrogen bonding interaction [38]. As can be observed in Figure S1, the hydrogen bond is highly affected by the dipole forces. Based on the DFT calculation, the obtained value showed an increment from 2.991912 D (amylose without water) to 3.193061 D (amylose with water). This change indicates some kind of dependency on the nature of the surrounding atom, in which the presence of an electronegative oxygen atom of O11 in the system yields a stronger inductive effect on the hydrogen atom of H111. On the other hand, the decreasing value of Mulliken charge for the oxygen atom of O11 in Figure S1b, and in the structure of amylose with and without water in Figure S1c, are −0.522e and −0.561e, respectively, proving the existence of a hydrogen interaction with H111 [39].

**Figure 5:**
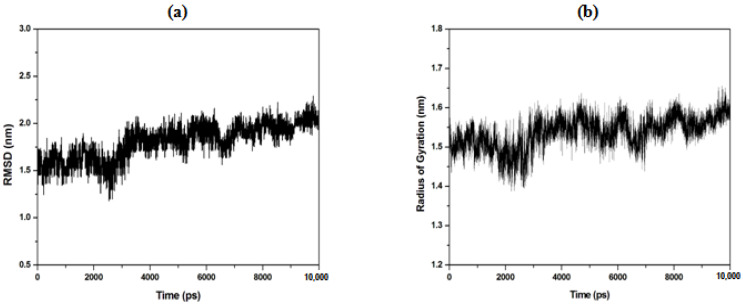
Molecular dynamics of (**a**) RMSD of amylose solvated in SPC water molecules at 298 K for 10 ns. The trajectory for analysis was taken from the last 2 ns of the NPT production simulation. (**b**) Average radius of gyration (RoG) of 50 molecules of amylose, representing the amylose region solvated in SPC water molecules at 298 K and 1.0 bar.

Furthermore, the interaction of starch with water was also analyzed using the molecular dynamics of both starch and water. The root mean square displacement (RMSD) measures the average atomic displacement between the initial and final conformations of the simulation trajectory. In this measurement, the initial conformation value was taken from the RCSB Protein Data bank (http://www.rcsb.org/ (accessed on 16 June 2020)), while the coordinate of the final position was obtained from the NPT production simulation. The structural stability of amylose solvated in water molecules was obtained from the last 2 ns, and the result is shown in Figure 5a. The analysis clearly shows that the average RMSD is about 0.18 Å, and this value is in good agreement with the report of a previous study [40]. This shows that the conformation structure of amylose had swelled and extended in the absence of an extrinsic mixture. It has been reported that in the presence of amylose–linoleic acid complexes, the ligand was able to preserve the helical conformation of the polymer [40,41].

Another analysis technique that can be used to study the formation of a hydrogen bond between starch and water is the radius of gyration (RoG). In this study, the RoG refers to the distribution of the atomic structure against its initial position. Figure 5b represents the RoG of amylose in water with an average value of 0.15 Å, which is slightly higher in comparison to the value reported by Gao et al. (2021) [42,43], who observed the RoG value of 0.13 Å for a single amylose strand. The obtained result indicated that the amylose fluctuated greatly and reduced its compactness in water at 298 K. When carbohydrates, which have hydrophilic properties, strongly interact with water via intermolecular bonding, the strong attraction between the carbohydrates and water will reduce the compactness of the amylose complex, since water molecules are highly mobile.

**Figure 6:**
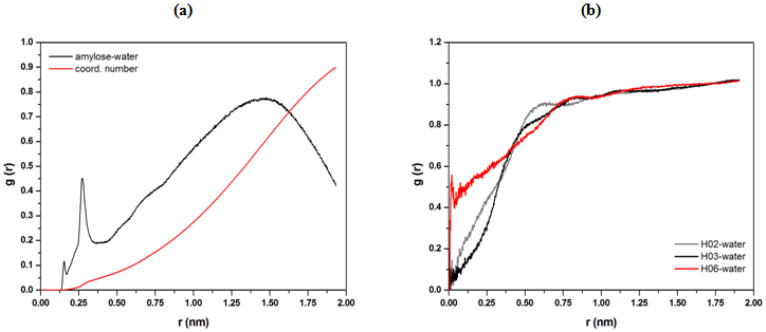
Center-of-mass radial distribution function (COM-RDF) of water molecules around the amylose structure (**a**) and specific COM-RDF of a hydrogen atom in amylose toward water molecules’ interaction (**b**). The g(r) represents the probability of finding an atom or molecule around a distance r of another chosen atom or molecule as a reference point.

The radial distribution function (RDF) describes the probability density of finding a molecule from a reference particle. In this study, the water density around the amylose molecule was investigated. Figure 6a shows that the RDF of the amylose water complex distance is smaller, with a distance value of about 2.6 Å. This validates our prediction on the existence of a hydrogen bonding interaction between the polar site of amylose with water molecules. The result indicates that water can penetrate the polymer networks, leading to a high probability of interaction with amylose molecules. This penetration causes the amylose structure to stretch out, thus decreasing its helical stability. The specific RDF atom–molecule interaction was also carried out to determine the parts in the amylose site wherein the most intermolecular hydrogen bonding interaction occurs. In general, amylose chains have a strong affinity towards themselves and towards materials that contain hydroxyl groups. The amylose chains are intra-connected via intra-molecular OH-O-type hydrogen bonds to form fleet sheets with CH-O hydrogen bonds [43]. The formation of intermolecular interaction is strongly dependent on the absorption sites: OH moieties pointing out of the amylose or above the equatorial hydroxyls. As shown in Figure 6b, the polar hydroxyl site of H06 was found to interact more with water molecules due to its flexibility and less steric hindrance as compared to other sites.

The average number of intermolecular hydrogen bonds between amylose and water molecules was determined from a production simulation trajectory. Based on the trajectory, a hydrogen bond is considered to exist in one conformation, provided that the distance between the hydrogen atom and the acceptor is less than 0.135 nm, and the angle formed by the acceptor–donor hydrogen is less than 30° [43,44,45]. In this study, the average number of hydrogen bonds calculated from the molecular dynamic (MD) simulation is 13, and this non-bonded interaction was expected to exist due to the polar OH group of amylose that provides sites for the hydrogen bond donor and acceptor. Most of the time, it can be assumed that the amount of hydrogen bonding was maintained throughout the simulation.

**Figure 7:**
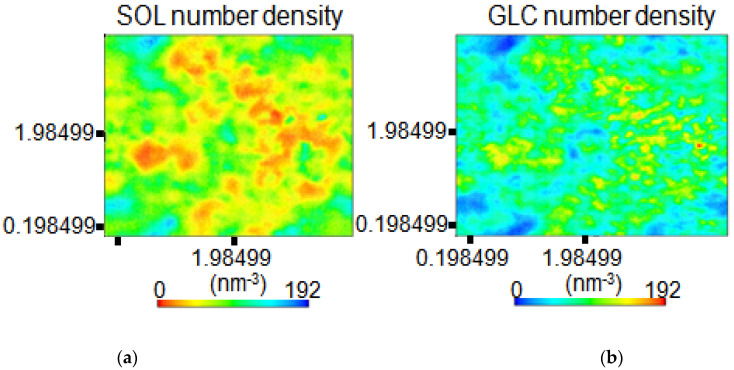
Density maps (number of molecules/nm^3^) showing (**a**) water molecule distributions and (**b**) amylose at P = 1.0 bar and T = 198.15 K (in the interlayer at d = 001 spacing) for the amylose–H_2_O system. The results were obtained by averaging over 2.0 ns of simulation time.

The number of density maps for water molecules residing in a polymeric cavity of amylose was obtained by scanning the axial space to compute the density distributions. The density distributions were projected onto the x-y plane and the simulation time was averaged for 2 ns. Based on the density maps shown in Figure 7, it was observed that the molecules were able to penetrate the amylose complex by splitting into smaller conglomerates, as indicated by the orange region. In addition, these density maps further confirm that the water molecules dissociated with the solvent as they interacted with amylose chains, thus reducing the hydrogen bonds among the amylose molecules.

### 3.3. Physiochemical Properties of S-SAP

**Figure 8:**
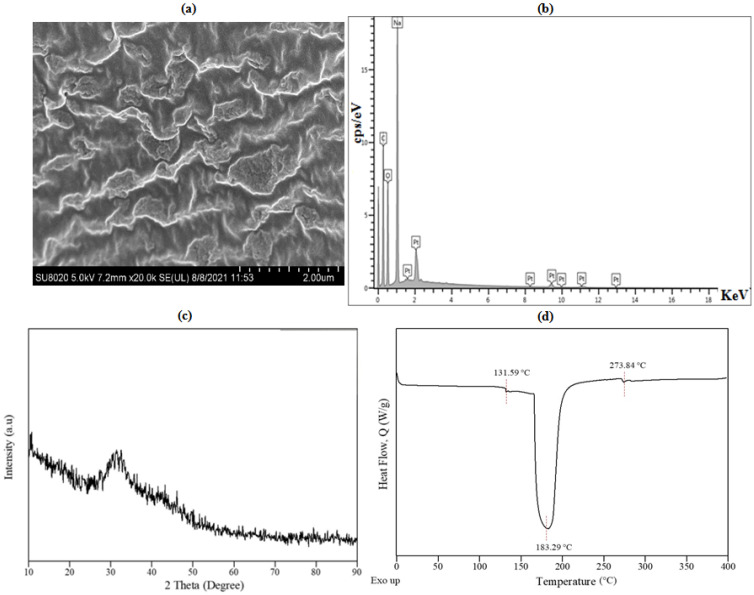
Analysis of S-SAP showing (**a**) FESEM micrographs at 20.0 k magnification, (**b**) EDX spectra for elemental composition of S-SAP, (**c**) XRD diffractogram, and (**d**) DSC thermogram.

The physicochemical properties S-SAP were studied using the FESEM, EDX, XRD and DSC analyses, as presented in Figure 8. Based on the micrograph shown in Figure 8a, it was observed that the morphology of S-SAP displayed continuous agglomeration of loose particles of homogeneous S-SAP. Based on the loose structure, it can be noted that the foaming process of the starch slurry assists in improving the SAP’s absorption characteristics. This finding is also in a good agreement with those reported by Saha et al. (2020) [46], in which the grafting of a compound onto the smooth and tight surface of the poly(acrylic acid) derivatives led to a heterogeneous and loose structure with several cavities present in the starch.

The elemental composition in the S-SAP was characterized using the EDX spectroscopy in Figure 8b. The successful formation of poly(methacrylic acid-co-sodium methacrylate) in the grafted S-SAP can be proven by the structural composition, as part of the methacrylic acid was neutralized after the addition of NaOH to form sodium methacrylate. The EDX analysis detected the presence of elements C, O and Na, with 52.3, 30.2 and 17.5 wt.%, respectively. These corresponded to the chemical composition of S-SAP, namely starch g-poly(methacrylic acid–co–sodium methacrylate). The diffraction pattern illustrated in Figure 8c shows that S-SAP was highly amorphous based on the appearance of wide and broad diffraction peaks. In Figure 8c, the appearance of wide and broad peaks at 2θ of 31.5° suggests that the prepared S-SAP was in an amorphous phase. In addition, the wide and low intense peaks at 2θ of 17° indicate the disappearance of the crystalline phase of starch [46]. The lack of intense sharp peaks suggests that the crystalline phase of starch was completely replaced during the graft polymerisation process, which involved gelatinization and the addition of poly(methacrylate-co-sodium methacrylate) blocks. 

The thermal stability and glass transition temperature (Tg) of S-SAP was studied using differential scanning calorimetry (DSC). The thermogram for SAP shown in Figure 8d was obtained using DSC that ramped up to 400 °C and a heating rate of 10 °C/min. As depicted in Figure 8d, the curves show three characteristic stages which denote the glass transition, melting and fusion of crystallite. The Tg occurred at 131.59 °C, followed by two endothermic curves which corresponded to endothermic transition at a peak temperature of 183.29 °C, and the fusion of crystalline at 274.23 °C with enthalpy of 889.19 J/g and 3.31 J/g, respectively. Figure 8d illustrates that the first endothermic curve in the DSC thermogram can be classified as an endothermic transition, which can be associated with the decreasing heat capacity of the sample. Moreover, weight loss was due to the loss of water that was tightly bound to the starch. Furthermore, the second endothermic peak, observed at 274.23 °C with enthalpy of 3.31 J/g, was detected as the fusion of crystallite, in which disintegrated S-SAP changed into small flakes and could be fused and combined together. 

**Figure 9:**
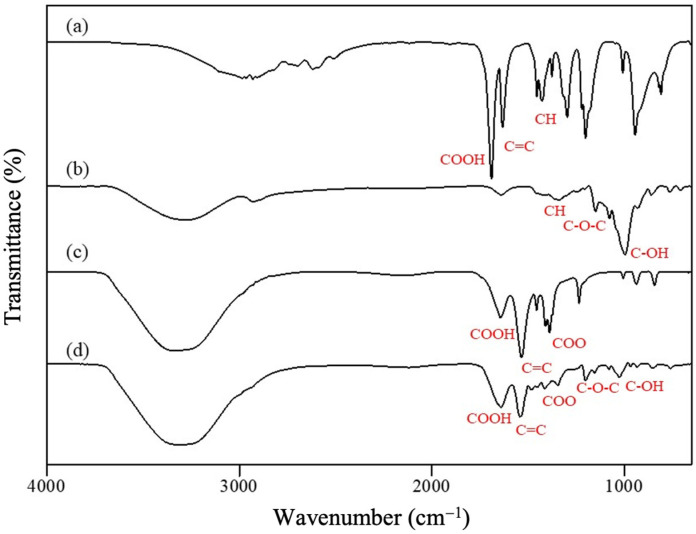
FTIR spectra of (a) methacrylic acid (b) starch (c) poly(methacrylic acid- co-sodium methacrylate) and (d) S-SAP.

The FTIR analysis was performed to identify the bonds and functional groups in the S-SAP ranging from 4000 to 650 cm^−1^. For comparison purposes, the FTIR analysis was also determined for methacrylic acid, starch, and poly(methacrylic acid-co-sodium methacrylate), along with SAP, to confirm the grafting of starch into poly(methacrylic acid-co-sodium methacrylate). Based on Figure 9, the peak observed at 1690 cm^−1^ in the methacrylic acid spectrum and at 1642 cm^−1^ in the SAP spectrum corresponded to the stretching vibration of the COOH group, thereby confirming the presence of poly(methacrylic acid-co-sodium methacrylate) in S-SAP. Meanwhile, the peaks at 1632, 1532, and 1541 cm^−1^ for the sample of methacrylic acid, poly(methacrylic acid-co-sodium methacrylate) and S-SAP, respectively, were attributed to C=C stretching vibrations [47]. Although the characteristic peaks of semi-crystalline pure starch around 1200–800 cm^−1^ had weakened, they were still present in SAP, confirming that the starch had grafted onto the poly(methacrylic acid-co-sodium methacrylate) networks.

### 3.4. The Performance of S-SAP

**Figure 10:**
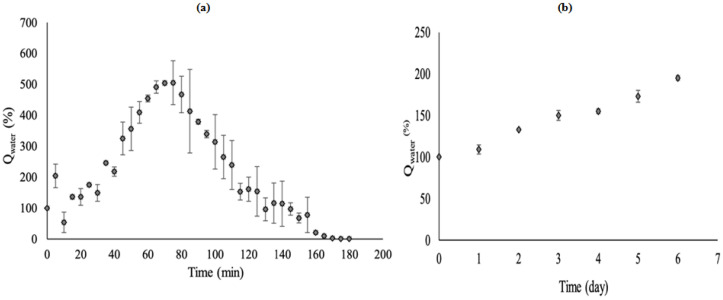
Difference in absorbency, Q_water_, of water versus time for (**a**) distilled water and (**b**) solid waste sludge.

The absorption properties of the S-SAP were investigated to determine the maximum absorbed water capacity and the performance of the polymeric materials. Figure 10 shows the absorption properties of S-SAP in both distilled water (as control) and solid waste sludge based on the differences in absorbance over time. As shown in Figure 10a, the water absorbency of S-SAP composite reflects three stages. The first stage (0–80 min) showed a rapid increase in the amount of water with time, while the second stage (80–160 min) revealed a decreasing absorption of distilled water by the superabsorbent. The final stage (165–180 min) depicted the absorbency almost in an equilibrium state, as no increase in water absorbency over time was recorded. The water absorbency of the S-SAP reached 500% after 80 min, but the absorption decreased subsequently and gradually in the second stage, i.e., from 500% to 100% for another 80 min (80–160 min) before reaching the equilibrium state at 0%. 

The rapid absorption of water in the first stage was due to the presence of numerous free OH groups in the polymeric network of the superabsorbent that are capable of forming hydrogen bonding with water. In the second stage, the OH group in the polymeric network was almost fully bonded with the free water molecules and thus was unable to absorb more water. This resulted in a decrease in the absorption efficiency of SAP. In the third stage, as all the OH groups in S-SAP were fully bonded to the water molecules, no hydrogen bond with water could be formed. Therefore, 0% of absorbency was plotted because the saturation point equilibrium was already achieved [48].

Figure 10b shows the absorption properties of S-SAP in solid waste sludge. The first stage of the absorption trend demonstrates that the amount of wastewater absorbed was increasing steadily up to 195% over 6 days. Compared to the absorption in distilled water, the absorption trend of wastewater from sludge was slower, since S-SAP absorbed the entrapped wastewater from damp sludge for the absorption to happen. In addition, the increased ionic strength of the wastewater due to its basic pH also caused a rapid decrease in ion osmotic pressure, which contributed to the slow uptake of wastewater from damp sludge by S-SAP. Based on the absorption trend of wastewater from the solid waste sludge, it is expected that the absorption of wastewater from sludge will continue until the saturation point of the S-SAP reaches 500%.

A preliminary study using the XRF spectroscopy was conducted to determine the efficiency of S-SAP in absorbing metal contaminants from solid waste sludge, as shown in Figure S2. The results show the dominant proportion of Na metal, with a concentration of 951000 ppm; this can be attributed to the sodium methacrylate at the end of the structure in SAP. In addition, ten metal elements were also detected alongside Na: Zn, Al, Si, S, Cl, K, Ca, Fe, Cu, and Zr. The preliminary results show that all the absorbed metals are positively charged, revealing that the absorption process occurred due to the electrostatic attraction between the positive metal ions and the negatively charged functional groups abundantly found in the S-SAP. The specific concentrations and details on the removal mechanism of the respective elements will be described elsewhere. 

**Figure 11:**
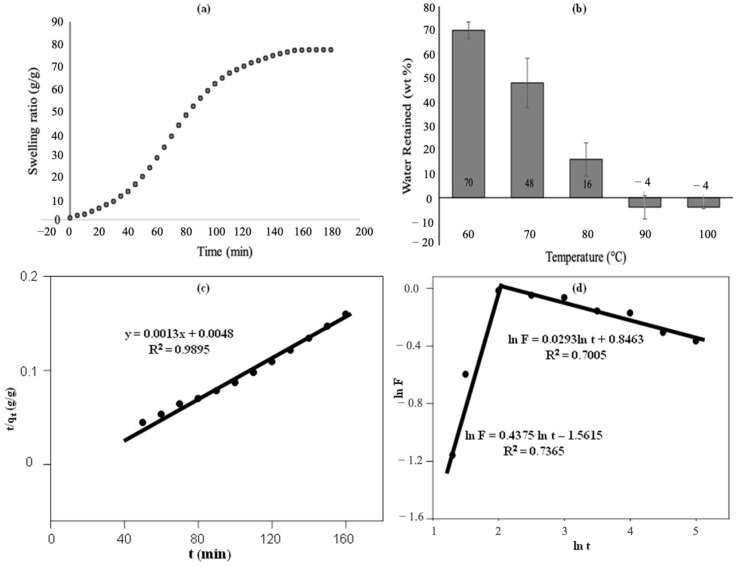
S-SAP performance for (**a**) swelling ratio (q) over time and (**b**) water retention at different temperatures, (**c**,**d**) swelling kinetics.

Figure 11a illustrates that the swelling of S-SAP occurred via two-stage processes of rapid swelling and steady-state/equilibrium state. During the rapid swelling process, the swelling ratio of S-SAP increased steadily at 160 min up to 77 g/g. At 160 min and above, the swelling ratio showed no changes, indicating that the maximum swelling capacity was achieved. The high swelling ratio of S-SAP observed early in the reaction can be explained through the available vacant area in the polymeric network that enables rapid penetration of the solvent into the superabsorbent. Therefore, the swelling ratio increased until the maximum capacity was achieved. Additionally, when a dry hydrogel is in contact with thermodynamically compatible solvent molecules, it will attack the hydrogel surface and penetrate into the polymeric network of the S-SAP [49]. More specifically, the expansion of the rubbery phase’s network resulted from the separation of the unsolvated glassy phase from the rubbery hydrogel region with a moving boundary. Hence, the vacant area was created during the expansion of the networks of the rubbery phase, thus enabling the molecules of solvent to enter the superabsorbent network.

Upon reaching the second phase (>120 min), the void in the polymeric network of S-SAP was almost filled with solvent molecules and completely filled when the saturation point of the S-SAP was reached (160 min). The stable three-dimensional structure of the prepared S-SAP resulted in a low swelling ratio, which was caused by high crosslinking density of the polymers. In particular, a higher concentration of crosslinkers produces a large number of growing polymeric chains that generate an additional network. Consequently, this results in a decreased swelling ratio when the crosslink density exceeds a certain degree, due to the decrease in space between the crosslink branching. However, the highly crosslinked S-SAP has a higher dry-state gel strength and can maintain its shape even after modest pressure. Ironically, the S-SAP does not have a good re-swelling capacity, which may be attributed to the irreversible collapse of the S-SAP during the re-swelling process at 110 °C for 90 min, in which the collapsed polymeric network suggests a low mechanical property. 

The ability of the superabsorbent to retain water content at a certain temperature was investigated by a water retention test. The test was performed by heating the swollen- S-SAP at different temperatures. The water retention capacity of S-SAP is presented in Figure 11b. The results show that the swollen superabsorbent has a decreasing tendency to retain water with increasing temperature. During the absorption process, the combination of the polymeric network of S-SAP opening up and the water molecules from the surrounding sticking up to it by forming hydrogen bonding causes the solid surface of the superabsorbent to become thicker and more viscous, thereby making the appearance of the superabsorbent sticky and swollen.

However, the hydrogen bonding formed between water molecules and the polymeric network is disrupted when the swollen polymer is exposed to high temperature. About 70% of water could be held at 60 °C after 5 h of the water retention test. Likewise, about 48% and 16% were retained at 70 °C and 80 °C after 5 h of heating. This shows that the S-SAP can hold water upon heating at 5 h, and implies that the superabsorbent possesses a good water retention ability and stable polymeric network even after the oven heating process. Moreover, the hydrogen bonding formed by the surrounding distilled water molecules with superabsorbent did not break down during the process. When the absorption process was conducted at humid temperature, the water would not easily release, even after being absorbed by the superabsorbent. 

According to previous works, the swelling behaviour of S-SAP was evaluated by the pseudo-second-order swelling kinetics model using Equation (3), and the Ritger–Peppas model using Equation (4):

(3) $$\frac{t}{q_{t}}=\frac{1}{k_{2}q_{e^{2}}}+\frac{t}{q_{t}}$$

(4) $$\ln F=\ln q_{t}-\ln q_{e}=\ln k+n\ln t$$

where $q_{e}$ (g/g) is the water absorbency at equilibrium and $q_{t}$ (g/g) is the water absorbency at time *t*. While $k_{2}$ (g∙mg^−1^ min^−1^) is the rate constant, *F* is the fractional uptake at time *t*, n is the swelling exponent, and k is the structural parameter.

As shown in Figure 11c, the relationship between $\frac{t}{q_{t}}$ and *t* can be considered linear, with a satisfactory correlation coefficient (R^2^ = 0.9895). The swelling kinetics of the S-SAP fit well with the pseudo-second-order relationship. Figure 11d shows an acceptable correlation coefficient of the Ritger–Peppas model which is almost comparable to similar works [49,50]. Based on this result, the chemical interactions via chemisorptions on S-SAP dominate the water absorption process.

In the case of high temperature heating at above 90 °C, the water retention value was negative, suggesting that the water released by heating is more than the absorbed water. This could be due to the partial vaporization of water content from the dry superabsorbent, along with the absorbed water upon 5 h of heating. Water molecules start to vaporize as they reach the boiling point of water. It should be noted that high temperature provides high kinetic energy to water molecules to break down the hydrogen bonding. In addition, the synthesized S-SAP could retain more than 50% of distilled water after 5 h of heating at 60 °C. For reusability, it is possible to determine the most suitable temperature for carrying out the desorption process and removing the water content from the S-SAP based on this information.

## 4. Conclusions

In summary, a new approach of using S-SAP for cost-effective solid waste sludge remediation is presented in this work. Firstly, the S-SAP was successfully prepared via graft polymerisation of poly(methacrylic acid-co-sodium methacrylate) as a monomer on the starch, using MBA as the crosslinker and APS as the radical initiator. The structural analysis showed that starch granules are semi-crystalline, as confirmed based on the XRD patterns. On consecutive addition of water to starch, the FTIR measured an intensified bending mode of H-O-H, suggesting water penetration into the starch polymer networks. Furthermore, the MD simulations assessed the formation of hydrogen bonding between starch and water on the polar hydroxyl site of H06 of amylose due to its flexibility and less steric hindrance as compared to other sites. Meanwhile, the DFT and MD simulation analyses showed that the starch backbone is highly capable of absorbing water efficiently into its polymeric network. Based on the MD simulation, the specific RDF atom–molecule interaction in the amylose backbone was able to record a high degree of water penetration into the S-SAP. By using DFT, the presence of hydrogen bonds can be confirmed by the changes in the value of Mulliken charge of atom O11. To support the simulation findings, the water retention of S-SAP was evaluated according to pseudo-second-order kinetics for chemisorption reactions.

The FESEM showed that the morphology of S-SAP possesses a continuous agglomeration of loose particles of homogeneous polymeric networks. Meanwhile, as confirmed by FTIR, the successful grafting of starch onto the PMA network might have decreased the crystallinity of semi-crystalline starch. The low crystallinity of S-SAP is in agreement with the XRD analysis. Absorption studies using water have shown that the S-SAP was capable of absorbing distilled water up to 500% (i.e., the saturation point) within 80 min. With respect to the absorption of water from solid sludge, the high amount of water absorption (up to 195%) and the significant water retention capacity were measured. In future, the S-SAP might have many potential applications as a low cost natural superabsorbent with a high water retention capacity, based on the presented water–polymer interaction.

## Figures and Tables

**Table 1:** Sample description of solid waste sludge.

Parameters	Description
Code	SW204
Origin	Pasir Gudang, Johor
Condition	Sludges containing one or several metals
Source	Polymer production plant
Crystallinity	Highly amorphous
Moisture contents	60–70 wt.%

## Data Availability

Not available.

## Supplementary Materials

The following supporting information can be downloaded at: https://www.mdpi.com/article/10.3390/polym15061471/s1. Figure S1 DFT analysis on (a) H-bond of O11 with H111. The Mulliken Charge of amylose without (b) water and (c) with water. Figure S2 XRF spectra for absorption of metals contaminants from sludge by NSS using (a) RX9 source, (b) Cu source, and (c) Mo source.
