# Supplementary material for: Starch-Derived Superabsorbent Polymer in Remediation of Solid Waste Sludge Based on Water–Polymer Interaction
Source: Polymers (Basel). 2023 Mar 16;15(6):1471. doi: 10.3390/polym15061471 (PMC10051928; doi:10.3390/polym15061471)
Supplement: Supplementary file 1 [file polymers-15-01471-s001.zip › polymers-2203470-supplementary.pdf]

polymers

MDPI

Article

Effect of Starch-Derived Superabsorbent Polymer on Water Retention and Absorbency Capability in Remediation of Solid Waste Sludge Based on Water/Polymer Interaction

Juan Matmin[1,6*], Salizatul Ilyana Ibrahim[2], Mohd Hayrie Mohd Hatta[3], Raidah Ricky @ Marzuki[1], Khairulazhar Jumbri[4], Nik Ahmad Nizam Nik Malek[5,6]

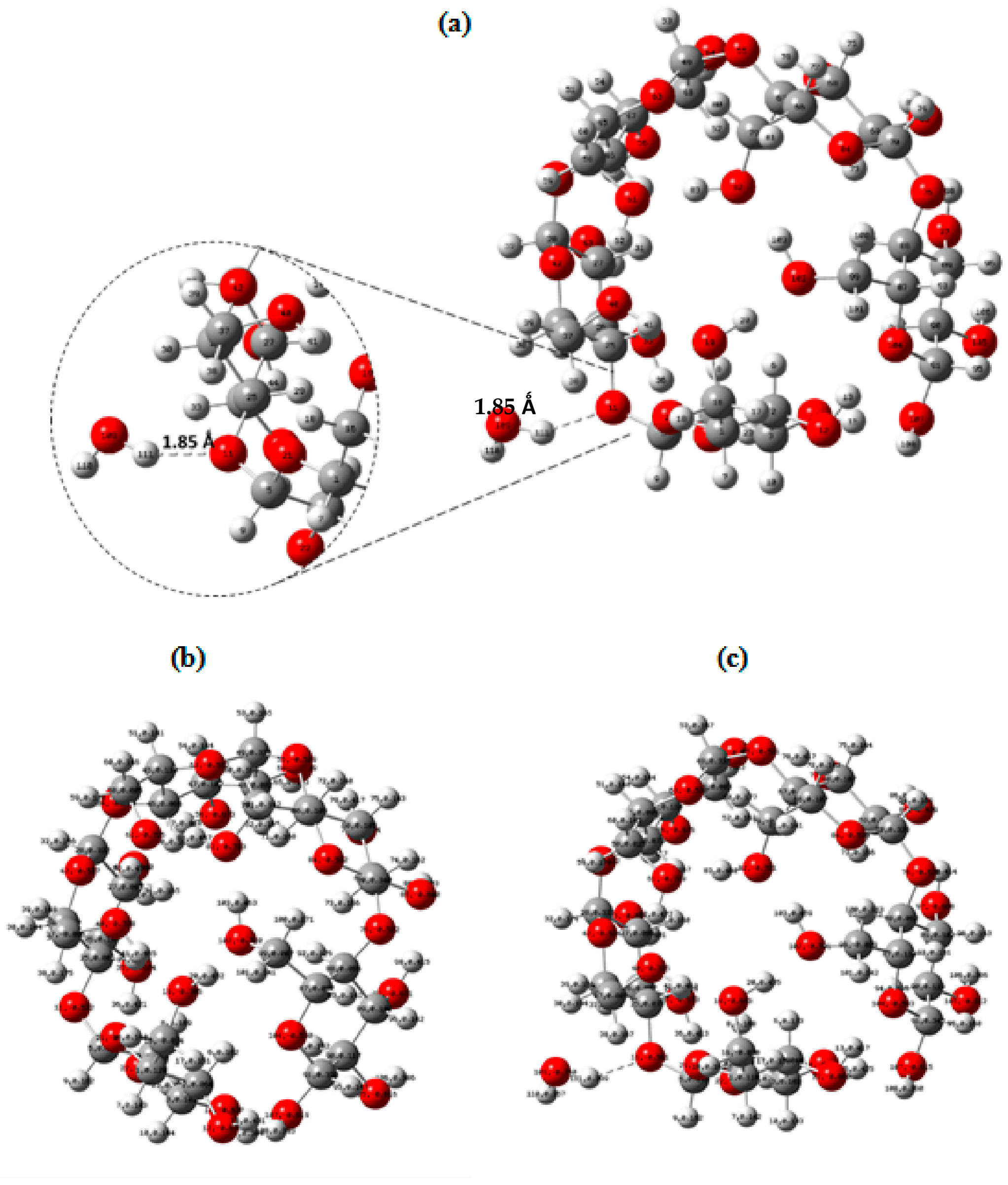

Figure S1 DFT analysis on (a) H-bond of O11 with H111. The Mulliken Charge of amylose (b) without water and (c) with water

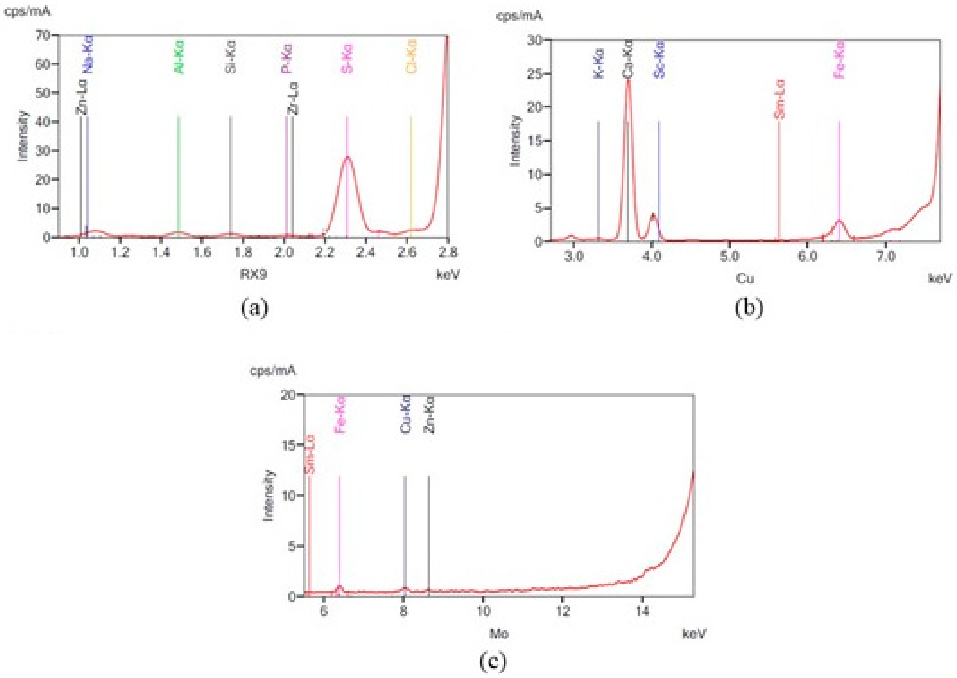

Figure S2 XRF spectra for absorption of metals contaminants from sludge by NSS using (a) RX9 source, (b) Cu source, and (c) Mo source.
